# Single-cell multiomic analysis reveals methylome and transcriptome deviations following oocyte maturation *in vitro*

**DOI:** 10.1530/REP-25-0011

**Published:** 2025-07-10

**Authors:** Camilla Benedetti, Carlo Giaccari, Francesco Cecere, Yannick Gansemans, Gavin Kelsey, Antonio Galvão, Simon Andrews, Nima Azari-Dolatabad, Andrea Fernandez-Montoro, Osvaldo Bogado Pascottini, Tine De Coster, Filip Van Nieuwerburgh, Trudee Fair, Erik Mullaart, Krishna Chaitanya Pavani, Ann Van Soom, Katrien Smits

**Affiliations:** ^1^ Department of Internal Medicine, Reproduction and Population Medicine, Faculty of Veterinary Medicine, University of Ghent, Merelbeke, Belgium; ^2^ Department of Environmental Biological and Pharmaceutical Sciences and Technologies (DiSTABiF), Università degli Studi della Campania “Luigi Vanvitelli”, Caserta, Italy; ^3^ Institute of Genetics and Biophysics (IGB) “Adriano Buzzati-Traverso”, Consiglio Nazionale delle Ricerche (CNR), Naples, Italy; ^4^ Laboratory of Pharmaceutical Biotechnology, Faculty of Pharmaceutical Sciences, Ghent University, Ghent, Belgium; ^5^ Epigenetics Programme, The Babraham Institute, Cambridge, United Kingdom; ^6^ Centre for Trophoblast Research, University of Cambridge, Cambridge, United Kingdom; ^7^ Wellcome-MRC Institute of Metabolic Science-Metabolic Research Laboratories, Cambridge, United Kingdom; ^8^ Department of Comparative Biomedical Sciences, Royal Veterinary College, London, United Kingdom; ^9^ Bioinformatics Unit, The Babraham Institute, Cambridge, United Kingdom; ^10^ Department of Animal Science, University of Tennessee, Knoxville, Tennessee, USA; ^11^ School of Veterinary Medicine, University College Dublin, Belfield, Dublin, Ireland; ^12^ School of Agriculture and Food Science, University College Dublin, Dublin, Ireland; ^13^ CRV, Arnhem, The Netherlands; ^14^ Department for Reproductive Medicine, Ghent University Hospital, Gent, Belgium

**Keywords:** developmental competence, mitochondria, imprinted genes, *in vivo* maturation

## Abstract

**In brief:**

*In vitro* maturation is an essential tool in reproductive technologies, though its impact on oocyte quality remains a concern. This study shows that *in vitro* maturation alters gene expression and DNA methylation in bovine oocytes compared to *in vivo* matured oocytes, potentially compromising oocyte quality and developmental competence.

**Abstract:**

*In vitro* maturation of oocytes is a routine step in assisted reproduction but is associated with lower embryo development rates compared to oocyte maturation *in vivo*. We analyzed the genomic profiles of oocytes from the same cow, either matured *in vivo* or *in vitro*, using single-cell methylome and transcriptome sequencing, along with transcriptome analysis of corresponding cumulus cells. Both the transcriptome and methylome of the oocytes matured *in vitro* were altered. Notable changes included alterations in CpG islands associated with imprinted genes, including decreased methylation levels in *MEST (PEG1), NNAT* (both implicated in large offspring syndrome), and *MIMT1*. Transcriptomic analysis of their cumulus cells highlighted impaired mitochondrial function, hypoxia responses, and cell adhesion. Our findings highlight the extent to which the maturation environment can influence key epigenetic regulators and mRNA profiles that affect oocyte quality and subsequent developmental outcomes. The data provide a valuable resource for optimizing assisted reproductive technologies.

## Introduction

During their growth and maturation within the follicular environment and their interaction with surrounding cumulus cells, oocytes acquire developmental competence, i.e. the ability to be fertilized and develop into an embryo (Eppig 1996). This competence is driven by the establishment of a specific epigenetic signature and accumulation of mRNA transcripts in the oocyte that support embryo development (Smallwood & Kelsey 2012). Immature oocytes from most species can resume meiosis once separated from their follicle environment and cultured within supportive culture media (Edwards 1965). *In vitro* maturation (IVM) is thus utilized for treating infertility in patients with polycystic ovarian syndrome, preserving fertility in cancer patients, and enhancing livestock breeding programs (Krisher 2022). However, IVM-derived oocytes show lower developmental competence, with reduced blastocyst rates (around 40%) compared to *in vivo*-matured (IVO) oocytes (around 60%, Rizos *et al.* 2002, Shani *et al.* 2023), and the mechanisms behind this decreased quality remain unresolved despite considerable investigation.

High-throughput technologies, such as RNA-seq, have been employed to determine the effect of the maturation environment on the cumulus–oocyte complex (COC). Initial studies in mice focused on the transcriptome of cumulus cells following IVM and IVO, concluding that cumulus cells fail to fully support oocyte maturation *in vitro* (Kind *et al.* 2013). More recent transcriptomic analyses in humans and bovines have identified a distinct messenger RNA (mRNA) signature in metaphase II (MII) oocytes compared to immature oocytes (Llonch *et al.* 2021, Latorraca *et al.* 2024), despite earlier indications that oocytes are transcriptionally silenced during maturation (Fair *et al.* 1996, Hyttel *et al.* 1997). It is likely that these discrepancies reflect dynamic changes in mRNA processing, storage, and degradation during maturation and ultimately the accessibility of mRNA during the isolation process (Pasternak *et al.* 2016, Cheng *et al.* 2022, Fair & Lonergan 2023). In mice and humans, transcriptome analysis showed that the expression of genes involved in mitochondrial, transcription, and cell cycle pathways was upregulated in IVM oocytes compared to those matured *in vivo* (Jones *et al.* 2008, Gao *et al.* 2017). However, these studies used pooled samples, potentially masking individual differences between oocytes. Equally important, the maturation environment may also alter the epigenetic landscape of oocytes. DNA methylation is the predominant epigenetic modification explored to understand problems derived from artificial reproductive technologies in humans and animals. It appears that various approaches to obtain mature oocytes exert a minimal effect on the DNA methylation profile (Saenz-de-Juano *et al.* 2019), but recent evidence suggests that the IVM condition, which is at present still suboptimal, may lead to changes in the oocyte epigenome (Ye *et al.* 2020). Therefore, analyzing the DNA methylation patterns of IVO oocytes may provide crucial insights into epigenetic anomalies observed in oocytes compromised by IVM procedures.

During postnatal growth, mammalian oocytes in meiotic arrest undergo *de novo* DNA methylation, which targets specific sequences, including differentially methylated regions (DMRs) of imprinted genes, defining their unique DNA methylation landscape (Smallwood & Kelsey 2012). In MII oocytes, the global DNA methylation level is predominantly intermediate, but quantitative differences were observed among species (Kobayashi *et al.* 2012, Smallwood & Kelsey 2012, Ivanova *et al.* 2020). Species-specific differences are present in the expression of *de novo* methyltransferase (DNMT) enzymes and zinc-finger proteins, which are crucial for maintaining DNA methylation at imprinted genes. In this regard, bovine oocytes mirror human gametes more closely than murine (Ivanova *et al.* 2020). In addition, the timing of embryonic genome activation in human embryos is also more similar to bovine than mouse (Sirard 2012). This activation could depend on or be associated with the process of maternal mRNA storage and degradation during oocyte growth and maturation (Cheng *et al.* 2022, Wu *et al.* 2023). In light of these observations, the cow represents a suitable animal model for humans to study epigenetic reprogramming and transcription occurring during oocyte maturation, particularly as the collection of IVO and IVM oocytes can be performed on the same animal, allowing for the individual donor effect to be taken into consideration during analysis.

The main objective of this study was to unravel the underlying molecular differences between *in vitro* and IVO bovine COCs. We used single-cell multi-omics analysis to evaluate the dynamics of gene regulation at the DNA methylation level and gene expression at the level of the mRNA transcriptome in MII oocytes following IVM or IVO from the same donor cows. Moreover, we performed transcriptome analysis on the corresponding cumulus cells to evaluate the effect of the maturation environment on the interaction between the somatic compartment and the oocytes. These results provide an important resource for a greater understanding of how IVM impacts oocyte competence and guide the optimization of protocols to enhance oocyte development and improve outcomes in both clinical settings and animal breeding programs.

## Materials and methods

### Animals

All experimental work at Coöperatie Rundveeverbetering (CRV) dairy breeding center was conducted under the Nederlandse Voedsel- en Warenautoriteit approval (license number 218336), with the permission of the Ethical Committee of the Faculty of Veterinary Medicine of Ghent University (EC 2020-082). Four non-lactating and non-pregnant Holstein heifers (15–18 months of age) were kept at the farm facilities at the CRV breeding center. The heifers were housed in group pens with ample space to move freely and had continuous access to clean drinking water. Their diet consisted of a balanced ration tailored to meet their nutritional needs, with regular veterinary check-ups to monitor their health and welfare. Animals were used for obtaining IVO COCs and collecting immature COCs through the ovum pick-up (OPU) procedure. The collection of IVO and IVM oocytes from the same cows was performed 30 ± 2 days apart. Animals were used between September 2021 and March 2022.

### Collection of IVO and VV bovine COCs

**Figure 1:**
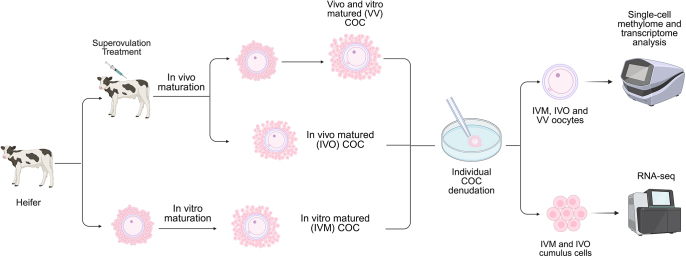
Visual representation of the experimental flow. Description of data: Heifers (four animals in total) were used to collect both IVO cumulus–oocyte complexes (COCs) and immature COCs subjected to IVM for 22 h. IVM and IVO oocytes were individually denuded from the cumulus cells to evaluate the maturation status, confirmed by the presence of the polar body. IVM, IVO, and VV oocytes were stored individually for further single-cell methylome and transcriptome analysis. Cumulus cells from mature oocytes were pooled and stored for further RNA analysis. Created with BioRender.com.

The experimental design is summarized in Fig. 1. Animals were synchronized using an intravaginal progesterone-releasing device (CIDR, Zoetis, Belgium). Eight days later, the device was removed, and heifers received 2 mL (0.5 mg) of prostaglandin (PG; Cyclix, Virbac, France). Estrus (=day 0) was confirmed 2 days later by ultrasound. To induce ovulation, 2.5 mL (10 μg) of gonadotropin-releasing hormone (GnRH, Receptal, Intervet, Germany) was injected on day 8 of the estrous cycle. From day 10, animals were superovulated with 180 mg of follicle-stimulating hormone (FSH, Folltropin, Vetoquinol, Canada) administered twice daily for 4 days in decreasing doses. On day 12, animals received 3 mL (0.75 mg) of PG, and the luteinizing hormone surge was induced with 2.5 mL GnRH administered 40 h after PG injection. Follicles ≥8 mm in diameter were aspirated 24 h after GnRH injection, using an 18-gauge needle. A 15–20 mL/min aspiration vacuum pressure was used due to the presence of expanded and sticky cumulus cells characteristic of oocyte maturation. Before follicle aspiration, the OPU tubing system was rinsed with polyvinylpyrrolidone (PVP) medium (0.3% PVP (PVP-360; Sigma, Belgium) in Ca- and Mg-free PBS + 10 IU/mL heparin (Sigma)). The follicular content was collected in a 50 mL conical tube, and COCs were recovered under a stereomicroscope and stored in EmXcell medium without BSA (IMV-Technologies, France) until the end of the collection procedure. After aspiration, COCs were collected under a stereomicroscope, and cumulus expansion was evaluated using a previously described scoring method (Downs 1989, Raes *et al.* 2023), where oocytes scoring 0–2 (poor expansion) were assigned to the VV group, and those scoring 3–4 (partial or full expansion) were included in the IVO group. Oocytes with evident cumulus expansion (IVO) were immediately denuded, whereas oocytes with poor cumulus expansion were submitted to IVM for 22 h (VV group) at 38.5°C in 5% CO2 in humidified air, in groups (based on the cow of origin), in maturation medium with 500 μL bicarbonate-buffered TCM199 medium supplemented with gentamicin (50 mg/L) and epidermal growth factor (20 μg/mL). To assess maturation status, IVO and VV COCs were denuded individually in 0.1% hyaluronidase (Sigma) in PVP medium for approximately 2 min and by pipetting with a STRIPPER pipette holder and a 135 μm capillary (Origio, Cooper Surgical, US) until all cumulus cells were removed from the oocyte. Denuded oocytes were classified as mature or immature oocytes based on the presence or absence of the polar body, respectively. Mature IVO oocytes (*n* = 29) and VV oocytes (*n* = 27) were washed individually three times in PVP medium, treated with pronase to dissolve the zona pellucida (0.1% protease from S. griseus in TCM-199), and immediately stored in 5 μL of Buffer RLT (Qiagen, Germany) at −80°C until further use. Cumulus cells from IVO COCs were collected and pooled based on the cow of origin and immediately stored in a lysis buffer for further analysis.

### Collection of IVM bovine COCs

Animals received GnRH (Receptal, Intervet, Germany) injection 8 days after estrus, and aspiration of follicles ≤8 mm was performed through an OPU procedure 4 days later. After aspiration, immature COCs (IVM group) were collected under a stereomicroscope and underwent IVM as described above. After 22 h, COCs were denuded, and mature oocytes (*n* = 36) with their respective cumulus cells were processed and stored as described in the previous paragraph.

### DNA and RNA isolation for single-cell bovine MII oocyte analysis

DNA and RNA from individual oocytes were separated using the G&T protocol described by Angermueller *et al.* (2016). Briefly, we used magnetic beads (MyOne C1, Life Technologies) annealed to Smart-seq2 oligo-dTs to capture polyadenylated mRNA from the single oocyte lysate. DNA from the remaining lysate was transferred to a separate tube, while the magnetic beads were washed three times in a solution containing 1×FSS buffer (Superscript II, Invitrogen, UK), 10 mM DTT, 0.005% Tween-20 (Sigma), and 0.4 U/μL of RNAsin (Promega, UK) to remove DNA residues. The washing solutions were combined with the DNA tube to maximize recovery. The mRNA attached to the magnetic beads was immediately processed for cDNA conversion by resuspending the beads in a 10 μL reverse transcriptase mastermix (100 U SuperScript II (Invitrogen), 10 U RNAsin (Promega), 1 × Superscript II First-Strand Buffer, 5 mM DTT (Invitrogen), 1 M betaine (Sigma), 9 mM MgCl2 (Invitrogen), 1 μM template-switching oligo (TSO, Eurogentec, UK), and 1 mM dNTP mix (Roche, UK)). After reverse transcription and amplification by PCR, the resulting product was purified using AMPure XP beads and eluted in 20 μL of water.

### Construction of scBS-seq libraries

The scBS-seq libraries were constructed following the protocol outlined previously (Clark *et al.* 2017). Briefly, DNA extracted from lysed cells was purified using a 0.9:1 ratio of AMPure XP beads (Beckman Coulter, UK) and eluted in 10 μL of water. Subsequently, single-cell DNA underwent bisulfite conversion using the EZ Methylation Direct Kit (Zymo, UK) as per the manufacturer’s guidelines. First-strand synthesis encompassed five rounds: initially, bisulfite-treated DNA was combined with 40 μL of first-strand synthesis master mix (1 × Blue Buffer (Enzymatics, UK), 0.4 mM dNTP mix (Roche), 0.4 μM 6NF oligo (IDT)), heated, and cooled before adding 50 U of Klenow exo–, followed by 37°C incubation. This process iterated four more times with an increased reaction mixture, concluding with a 90 min incubation. Exonuclease digestion ensued by introducing 20 U of exonuclease I (NEB) in a 100 μL volume at 37°C for 1 h. The resultant samples underwent purification using AMPure XP beads with a 0.8:1 ratio. Subsequently, a second-strand master mix (1× Blue Buffer (Enzymatics), 0.4 mM dNTP mix (Roche), 0.4 μM 6NF oligo (IDT)) was combined with the beads and processed through a heating–cooling cycle before adding 50 U of Klenow exo- and incubating at 37°C. The resulting samples underwent further purification with a 0.8:1 ratio of AMPure XP beads. Libraries were amplified using a 50 μL PCR master mix (1× KAPA HiFi ReadyMix, 0.2 μM PE1.0 primer, 0.2 μM iTAG index primer). Amplification involved an initial step at 95°C for 2 min, followed by 14 cycles of 80 s at 94°C, 30 s at 65°C, and 30 s at 72°C, concluding with an extension of 3 min at 72°C. The scBS-seq libraries were purified using a 0.7:1 ratio of AMPure XP beads and eluted in 15 μL of water. Pooled libraries were sequenced, with 48-library pools sequenced on an Illumina HiSeq 2,500 platform, generating an average of 13.0 million paired-end reads with a read length of 75 bp.

### Construction of scRNA-seq libraries

The mRNA attached to the magnetic beads was immediately processed for cDNA conversion by resuspending the beads in a 10 μL reverse transcriptase mastermix (100 U SuperScript II (Invitrogen), 10 U RNAsin (Promega), 1 × Superscript II First-Strand Buffer, 5 mM DTT (Invitrogen), 1 M betaine (Sigma), 9 mM MgCl2 (Invitrogen), 1 μM template-switching oligo (TSO, Eurogentec), and 1 mM dNTP mix (Roche)). The mRNA mixture underwent reverse transcription through a series of incubation steps: 60 min at 42°C, followed by 30 min at 50°C, and then 10 min at 42°C. The resulting cDNA was amplified by adding 11 μL of 2× KAPA HiFi HotStart ReadyMix and 1 μL of ISPCR primer (2 μM). The amplification process consisted of an initial step at 98°C for 3 min, followed by 15 cycles of 98°C for 15 s, 67°C for 20 s, and 72°C for 6 min, with a final extension at 72°C for 5 min. After reverse transcription and amplification by PCR, the resulting product was purified using AMPure XP beads and eluted in 20 μL of water. Libraries were created from 100 to 400 pg cDNA using the Nextera XT Kit (Illumina, UK) following the manufacturer’s instructions, albeit with one-fifth volumes. Finally, a pool of all 88 single-cell RNA-seq libraries was sequenced on an Illumina NextSeq platform, averaging a depth of 4.2 million reads, employing paired-end 75 bp read-length settings.

### Total RNA extraction and transcriptome analysis of cumulus cell samples

Total RNA was extracted from nine CC samples (four from IVO, five from IVM COCs) using the RNeasy Micro Kit (Qiagen) according to the manufacturer’s instructions. The RNA samples underwent quality and concentration assessments using an RNA 6000 Pico Chip (Agilent Technologies, USA) and a Quant-iT RiboGreen RNA Assay Kit (Life Technologies, USA), respectively. Following quality control checks for total RNA (evaluating RNA quantity and RIN value), four CC samples from the IVO group and three from the IVM group were used for further analysis. For each sample, a sequencing library was constructed using a SMART-seq3 protocol. This technology targets the 5′ end of the original mRNA. It incorporates an 11-base tag, followed by an 8-base unique molecular identifier (UMI) and two additional A/T at the 5′ end of the main R1 read, allowing identification of PCR duplicates and elimination of amplification bias. The libraries were pooled equimolarly, spiked with 20% PhiX, and sequenced as paired-end 76 on a NextSeq 500 device (Illumina). UMI, tag, and spacer were removed from the raw sequencing reads using UMI-tools (v1.1.4) (Smith *et al.* 2017). Adapter trimming was done using Cutadapt (v4.4) (Martin 2011), with added filtering of reads containing ambiguities or not passing the Phred score threshold of 20. Trimmed reads were mapped on the cow genome (UMD3.1.1) using the splice-aware STAR (v2.7.10a) mapper (Dobin *et al.* 2013). UMI-based deduplication of mapped reads was done with UMI-tools (v1.1.4). Feature counting at the gene and transcript isoform level was done using rsem-calculate-expression (RSEM v1.3.3) (Li & Dewey 2011). Data processing was done in R (v4.2.2) using the edgeR (v3.38.4) package for differential gene expression analysis (Chen *et al.* 2016).

### Processing of scRNA data

We processed a total of 88 libraries, adapting and quality-trimming them using Trim Galore version 0.4.4 (https://www.bioinformatics.babraham.ac.uk/projects/trim_galore) for sequences with a Phred score of less than 20. In addition, one negative control was sequenced to detect any technical issues. High-quality reads were aligned to the bovine reference genome (UMD3.1.1) using HiSat2 version 2.1.0 in single-end mode. The sequencing data were quantified with SeqMonk and subsequently analyzed in RStudio. After filtering out the genes not covered and the samples with low coverage, we used the 79 remaining cells (IVM = 32, IVO = 27, and VV = 20) to perform the differential expression analysis using the DESeq2 pipeline (Love *et al.* 2014). To delete any bovine-specific effect in the differentially expressed genes (DEGs), we put inside the DESeq model also the factor that identifies the bovine from which the oocytes were kept. We imported the list of DEGs into the STRING database (Jensen *et al.* 2009) and conducted cluster analysis using the ‘k-mean clustering’ option with the ‘number of clusters’, where the edges between clusters were depicted as dotted lines. To investigate the biological processes involving these proteins, we utilized gprofiler2 (Kolberg *et al.* 2020) with default settings. Gene ontology analysis was performed using ShinyGO 0.77 as previously described.

### Processing of scBS-seq data

A total of 46 (IVO = 20, IVM = 26) scBS-seq libraries were processed for analysis. The initial six base pairs, which included the N portion of the random primers, adapters, and low-quality bases (Phred score <20), were trimmed using Trim Galore version 0.4.4 (https://www.bioinformatics.babraham.ac.uk/projects/trim_galore) in single-end mode. High-quality reads were aligned to the bovine reference genome (UMD3.1.1) using Bismark version 0.18.2 (Krueger & Andrews 2011) in single-end and nondirectional mode, followed by deduplication and methylation calling through Bismark functions. MII scBS-seq libraries with insufficient mapping efficiency or a low number of covered CpGs were excluded from the analysis. In addition, samples with potential somatic DNA contamination, identified by an X-chromosome CpG island mean methylation above 12.5%, were removed (Castillo-Fernandez *et al.* 2020). The bisulfite methylation pipeline in SeqMonk was used to calculate the methylation profile for each feature. To assess global methylation differences between IVM and IVO MII oocytes, we calculated 100-CpG tiles using SeqMonk. CGI was downloaded from the UCSC Genome Browser. Gene body, promoter regions (gene upstream: −1,000 to +100), and other methylation features were quantified using the bisulfite pipeline in SeqMonk. Coordinates for imprinted gDMRs were built manually by referencing the gene locations provided at (http://www.geneimprint.com/). Gene ontology analysis was performed with ShinyGO 0.77, as previously described.

## Results

### *In vitro* maturation induces alterations in oocyte transcriptome and methylome, indicative of compromised oocyte maturation

**Figure 2:**
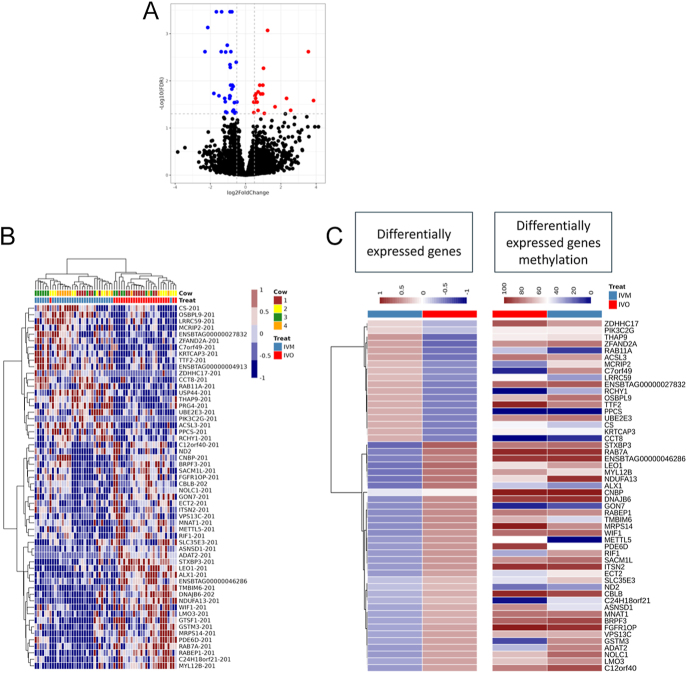
Single-cell RNA-seq analysis of MII bovine oocyte after *in vivo* (IVO) or *in vitro* (IVM) maturation. Description of data: (A) Volcano plot representing the DEGs between IVO and IVM MII oocytes. The x-axis of the graph reports the log2FC, while the y-axis represents the significance level denoted by −log10 (FDR). The dashed lines delineate the thresholds for discerning DEGs with FDR < 0.05. (B) Heatmap displaying the DEGs between IVM and IVO MII oocytes. Each column represents a distinct sample, with accompanying data denoting the corresponding cow origin. Rows represent the different genes, and hierarchical clustering delineates their segregation into two clusters corresponding to the two maturation sites (IVM and IVO). (C) Heatmap comparing the DEGs with corresponding methylation levels. Rows represent the different genes, and hierarchical clustering delineates their segregation into two clusters corresponding to the two maturation sites (IVM and IVO). The expression profile is represented as scaled FPKM.

To examine the impact of IVM on global gene expression and DNA methylation in oocytes, we utilized single-cell methylome and transcriptome sequencing (scM&T-seq) (Angermueller *et al.* 2016). Detailed information regarding the sequencing output of single-cell RNA-seq and bisulfite sequencing (BS-seq) libraries is provided in Supplementary Tables 1 and 2 (see section on Supplementary materials given at the end of the article). Comparison of IVM (*n* = 32) and IVO (*n* = 27) oocytes collected from four heifers resulted in the identification of 56 DEGs (Padj <0.05) (Supplementary Table 3). Of these, 35 were downregulated, and 21 were upregulated in the IVM group (Fig. 2A). Hierarchical clustering delineated segregation of the IVM and IVO oocytes (Fig. 2B). A STRING k-mean cluster analysis indicated 15 DEGs, forming four clusters associated with i) myosin binding, ii) NADHX epimerase activity, iii) ATP-dependent chromatin remodeler activity, and iv) phosphatidylinositol-4-phosphate phosphatase activity (Supplementary Fig. 1).

**Figure 3:**
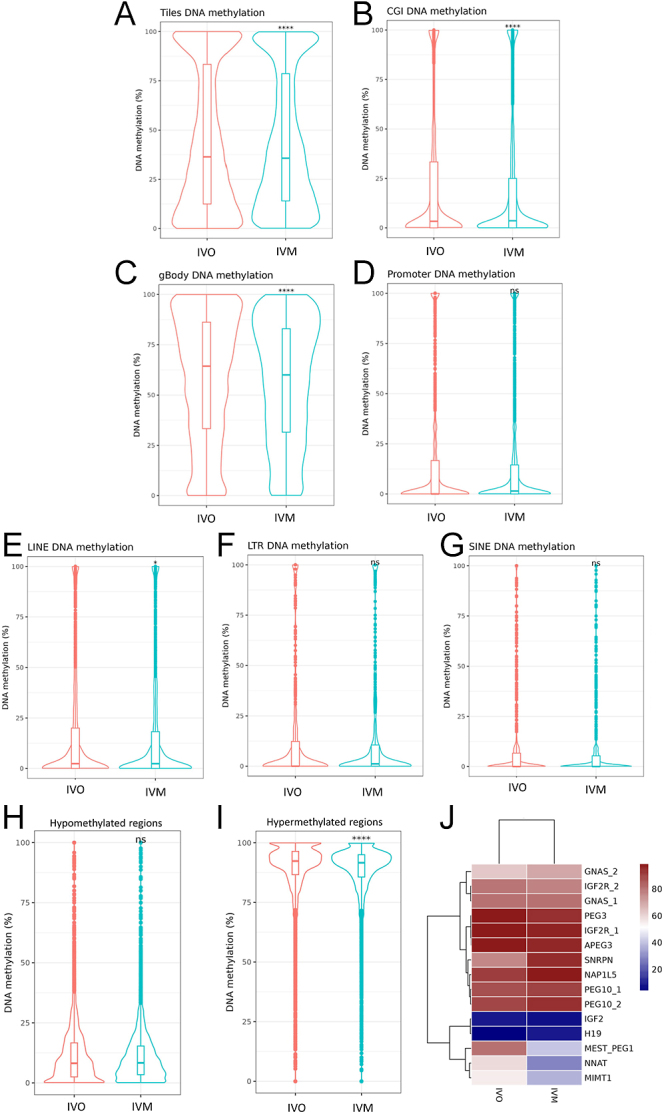
Single-cell bisulphite-seq analysis of MII bovine oocyte after *in vivo* (IVO) or *in vitro* (IVM) maturation. Description of data: (A) Violin plot displaying the percentage of DNA methylation of the whole genome divided into 100-CpG tiles. (B) Violin plot showing the mean percentage of CpG methylation at CpG islands (CGI) of MII oocytes after IVM or IVO, assessed by single-cell bisulfite and passing quality control. (C and D) Violin plots displaying the DNA methylation levels at gene bodies and promoter regions, respectively (TSS−1,000 +100). (E, F, G) Violin plots representing CpG methylation distribution across DNA repeated regions, including LINE, LTR, and SINE. (H and I) Violin plots of hypo- (H) and hyper-methylated regions (I) in IVM compared to IVO MII oocytes. (J) Heatmap displaying the imprinted germline differentially methylated regions (gDMRs) between IVM and IVO MII oocytes analyzed as average methylation between the two groups. Rows represent the different genes, and hierarchical clustering delineates their segregation into two clusters corresponding to the two maturation sites (IVM and IVO). The data shown in A–I were analyzed using Wilcoxon signed-rank test: *P* > 0.05 (ns), *P* < 0.05 (*), *P* < 0.01 (****).

**Figure 4:**
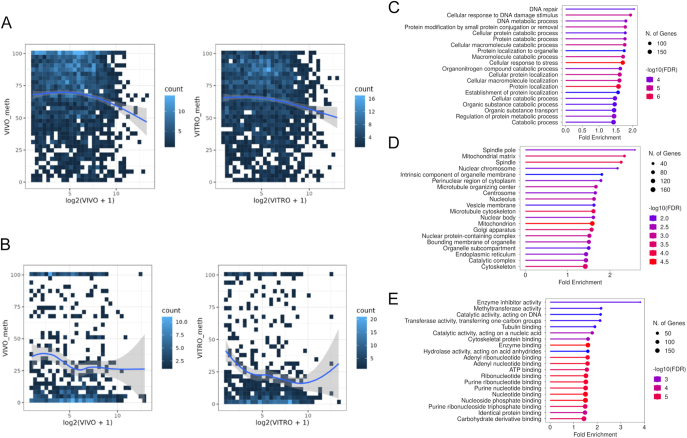
Correlation of CpG methylation and gene expression in bovine MII oocytes after *in vitro* (IVM) and *in vivo* (IVO) maturation. Description of data: (A and B) correlation of DNA methylation and gene expression of gene body (A) and promoter (B). The x-axis represents the gene expression levels, with log2 (*vitro* + 1) for IVM oocytes and log2 (*vivo* + 1) for IVO oocytes, while the y-axis represents the DNA methylation levels. (C, D, E) GO enrichment of DMRs of regions over 80% methylation. GO analysis of biological process (C), cellular component (D), and molecular function (F) were performed with ShinyGO 0.77 using the protein-coding genome as background (Ge *et al.* 2020) to identify enriched pathways (FDR < 0.05).

Single-cell bisulfite sequencing (scBS-seq) indicated hypomethylation in the whole–genome profile for the IVM group relative to the IVO (Fig. 3A). We observed significant changes in the DNA methylation distribution in CpG islands (CGI) and gene bodies (Fig. 3B and C; Supplementary Tables 4 and 5), but no significant differences in the promoter regions (Fig. 3D; Supplementary Table 6) between IVO and IVM oocytes. The methylation distribution across DNA repeat regions, including long interspersed nuclear elements (LINEs), short interspersed nuclear elements (SINE), and long terminal repeat (LTR) sequences showed limited changes in the LINEs (Fig. 3E; Supplementary Table 7), but no significant differences in LTR (Fig. 3F; Supplementary Table 8) and SINE (Fig. 3G; Supplementary Table 9) between IVO and IVM oocytes. We further examined the hypo- and hypermethylated domains that had previously been characterized in bovine MII oocytes (Ivanova *et al.* 2020). This analysis found no difference in hypomethylated domains between IVO and IVM oocytes (Fig. 3H; Supplementary Table 10), but identified significant hypomethylation within hypermethylated domains in IVM oocytes compared to IVO oocytes (Fig. 3I; Supplementary Table 10). Moreover, only 15 of the imprinted germline DMRs (gDMRs) identified in bovines, as listed on (http://www.geneimprint.com/site/genes-by-species.Bos+taurus), were covered by the captured CpG islands (CGIs) using scBS-seq. Three maternally imprinted genes, including Mesoderm specific transcript (*MEST*), Neuronatin (*NNAT*) and MER1 repeat containing imprinted transcript 1 (*MIMT1*), had lower DNA methylation in IVM than IVO oocytes, while Small nuclear ribonucleoprotein polypeptide N (*SNRPN*) was more highly methylated in IVM than IVO oocytes (Fig. 3J; Supplementary Table 11). A high-level comparison of the overall gene expression and the gene body or promoter methylation showed similar patterns for both IVO and IVM oocytes (Fig. 4A and B). At the gene body level, we observed a trend suggesting a negative correlation between methylation and gene expression (Fig. 4A), but overall, no significant correlation was found. Similarly, there was no correlation between DEG mRNA expression and DMRs (Fig. 2C; Supplementary Table 12). GO analysis of gene body DMRs – specifically those with 80–100% hypermethylated tiles – identified between IVM and IVO oocytes revealed that ≥10% were significantly enriched in several processes, including DNA repair, macromolecule catabolic process, protein localization, enzyme inhibitor activity, and ATP binding (Fig. 4C, D, E). Conversely, analysis of gene body hypomethylated tiles (0–20%) did not identify significant differences. Overall, our analysis showed that oocytes matured *in vitro* have distinct epigenetic characteristics compared to those matured *in vivo*.

### *In vivo* initiation followed by *in vitro* completion of maturation affects the oocyte mRNA profile

**Figure 5:**
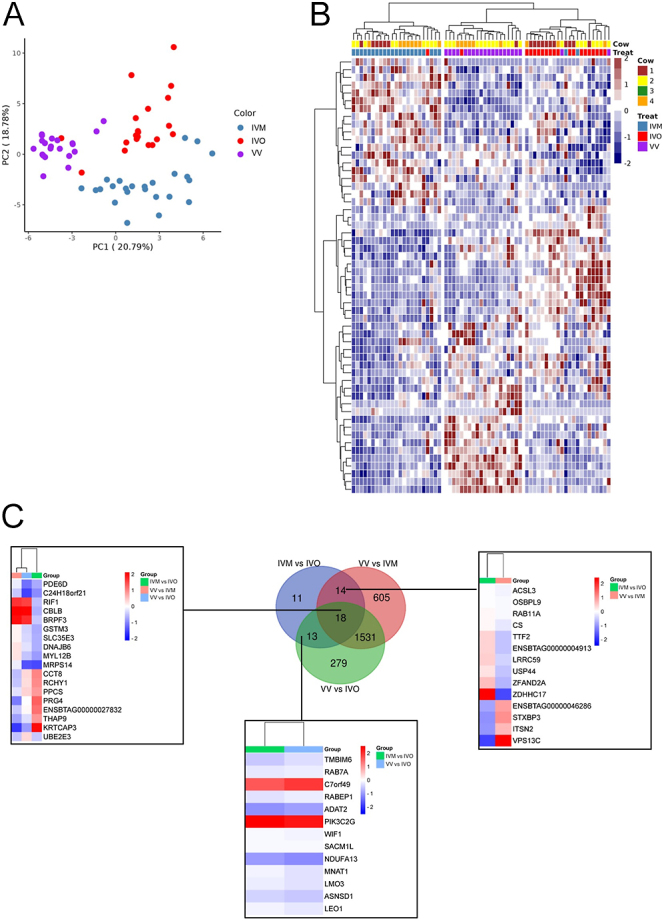
Single-cell RNA-seq analysis of MII bovine oocyte after *in vivo* maturation (IVO), IVM, or maturation initiated *in vivo* and completed *in vitro* (VV). Description of data: (A) PCA plot of the complete dataset covered by the RNA-seq experiment. The first principal component (PC1) is on the x-axis, while the second principal component (PC2) is on the y-axis. Each dot represents one MII oocyte. (B) Heatmap displaying the DEGs between VV, IVM, and IVO MII oocytes. Each column represents a distinct sample, with accompanying data denoting the corresponding cow origin. Rows represent the different genes, and hierarchical clustering delineates their segregation into three clusters corresponding to the three maturation sites (IVM, IVO, or VV). The expression profile is represented as scaled FPKM. (C) Venn graph showing the common DEGs between IVM vs IVO, VV vs IVM, and VV vs IVO.

After the IVO collection protocol, IVO COCs with no cumulus expansion (*n* = 27; from two cows) were cultured in maturation medium for 22 h. Once polar body presence was confirmed (85% maturation rate; MII oocytes = 23; four oocytes failed to mature), these MII oocytes were categorized as ‘*vivo vitro* (VV)’ and submitted to single-cell RNA-seq (scRNA-seq). Principal component analysis and hierarchical clustering examination showed a trend of clustering of VV between IVM and IVO (Fig. 5A and B). Comparison of VV vs IVM oocytes revealed 2,169 DEGs (1,230 down- and 939 up-regulated; Supplementary Table 13), and 1,841 DEGs (1,079 down- and 762 up-regulated; Supplementary Table 14) were identified in VV compared to IVO. In VV vs IVM, GO clusters were enriched in cellular response to stress, cellular catabolic process, transcription corepressor activity, and ubiquitin protein ligase binding (Supplementary Fig. 2). The comparison between VV vs IVO oocytes indicated altered pathways involved in cellular response to stress, organelle organization, protein N-terminus binding, and ubiquitin protein ligase binding (Supplementary Fig. 3). We identified 605 unique DEGs in VV vs IVM (Fig. 5C; Supplementary Table 15), while 279 DEGs were unique in VV vs IVO (Fig. 5C; Supplementary Table 16). A total of 18 DEGs were common among all the comparisons (IVM vs IVO, VV vs IVM, and VV vs IVO), 13 DEGs were shared between VV vs IVO and IVM vs IVO, and 14 DEGs were shared between VV vs IVM and IVO vs IVM (Fig. 5C).

### *In vitro* maturation alters the gene expression of cumulus cells

**Figure 6:**
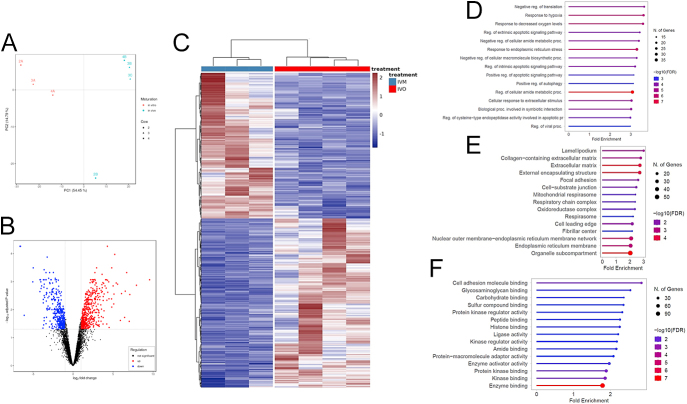
Gene expression profile of cumulus cells collected from cumulus-oocyte complexes after *in vitro* (IVM) and *in vivo* (IVO) maturation. Description of data: (A) PCA plot of the complete dataset covered by the RNA-seq experiment. The first principal component (PC1) is on the x-axis, while the second principal component (PC2) is on the y-axis. (B) Volcano plot representing the DEGs between IVO and IVM cumulus cells. (C) Heatmap generated by clustering the DEGs in IVO and IVM cumulus cells. Red, upregulation; blue, downregulation compared to the mean expression over all samples. (D, E, F) GO enrichment of DEGs. GO analysis was performed with ShinyGO 0.77 using the protein-coding genome as background (Ge *et al.* 2020) to identify enriched pathways (FDR < 0.05).

We compared the gene expression profile of pooled cumulus cells from IVM (three replicates; nine COCs per replicate) and IVO (four replicates, six COCs per replicate) MII oocytes, using RNA-seq. The PCA revealed a distinct separation of IVM and IVO samples into two clusters (Fig. 6A). We identified 844 DEGs (Supplementary Table 17), of which 454 were up- and 390 were downregulated in IVO compared to IVM (Fig. 6B). Hierarchical clustering analysis validated the classification of DEGs into IVM and IVO, as visualized in the heatmap (Fig. 6C). Enrichment analysis revealed GO clusters enriched in response to hypoxia and regulation of extrinsic apoptotic signaling pathway for biological function, cell adhesion molecule binding, glycosaminoglycan binding, and carbohydrate binding for molecular function, and lamellipodium, extracellular matrix, and oxidoreductase complex in cellular function (Fig. 6D, E, F). To investigate the impact of IVM on cumulus cell function, we performed STRING k-mean cluster analysis on the 454 downregulated genes in IVM. These genes were sorted into seven functional clusters, including electron transport chain, mitochondrial respiratory chain complex I assembly, and receptor signaling via STAT (Supplementary Fig. 4), indicating impaired mitochondrial activity in IVM-derived cumulus cells.

## Discussion

This study reveals that IVM can significantly alter the epigenetic and mRNA profile of mature oocytes, potentially compromising their quality. Most DEGs were downregulated in IVM oocytes compared to IVO oocytes. This finding is consistent with previous studies that have associated IVM with a decrease in mRNA levels (Caetano *et al.* 2023). Although *de novo* mRNA synthesis is quiescent following oocyte nucleus breakdown, IVM has been associated with an altered mRNA profile in matured oocytes (Mamo *et al.* 2011, An *et al.* 2024). These modifications are related to the degradation and polyadenylation of mRNA transcripts accumulated during the oocyte growth phase. The correct balance between mRNA degradation and storage during maturation shapes oocyte competence, thus determining the success of embryo development (Pasternak *et al.* 2016, Cheng *et al.* 2022). Yet, the downregulation of mRNAs observed in IVM oocytes suggests a significant impact on the transcriptomic landscape, potentially affecting the availability of critical transcripts to support oocyte competence and subsequent embryo development.

In our study, IVM of bovine oocytes led to relatively minor transcriptome modifications, with only 56 DEGs compared to IVO. In contrast, in human and mouse, significant differences in gene expression were found among IVO and IVM oocytes (Ye *et al.* 2020, Takashima *et al.* 2021). Notably, our bovine oocytes completed the growth phase within an optimized hormonal environment through the use of an estrus synchronization protocol, which is typically not employed in human and murine studies. This methodological difference may contribute to the observed discrepancies in gene expression. To ensure comparability in maturation conditions, we aimed to collect oocytes from follicles of different sizes. Specifically, oocytes from 3 to 8 mm follicles, which are typically associated with competent immature oocytes (Hyttel *et al.* 1997), were used for IVM. For IVO, we selected larger follicles (over 8 mm) that may have already undergone hormonal stimulation, leading to meiotic resumption and *in vivo* maturation (Hyttel *et al.* 1986). Moreover, as oocytes increase in size and reach the final growth stage, few changes occur in gene expression, with the most dynamic transcription phase occurring just before oocyte collection from the 3 to 8 mm antral follicles (Latorraca *et al.* 2024). Therefore, our results likely reflect the stabilized gene expression profile of fully grown oocytes from mid-antral follicles. Despite this, our findings align with those in human studies regarding the pathways affected in IVM oocytes, specifically showing that the pathways involved in NADH production were compromised in IVM oocytes in both human and bovine (Zhao *et al.* 2019). Among the downregulated DEGs in IVM, we identified three genes of interest. The first was *METTL5*, a methyltransferase essential for ribosomal RNA methylation and transcript translation (Turkalj & Vissers 2022). Its absence in mouse embryonic stem cells reduces translation, impairs pluripotency, and hinders differentiation (Ignatova *et al.* 2020), suggesting that IVM conditions may negatively affect genes critical for oocyte and early embryo totipotency. Next, we identified two genes involved in mitochondrial function. NADH dehydrogenase 2 (*ND2*) and NADH ubiquinone oxidoreductase subunit A13 (*NDUFA13*) encode two subunits of mitochondrial complex I and are involved in the mitochondrial electron transport chain (Janssen *et al.* 2006). Mitochondria are abundantly present in oocytes, playing a crucial role in oocyte maturation by providing ATP. A defect in *ND2* expression has been reported in immature oocytes of aged mice (Zhang *et al.* 2019), while *NDUFA13* expression decreases as bovine oocytes progress from immature to mature stage *in vitro* (Reyes *et al.* 2015). Both genes (ND2 and NDUFA13) play a crucial role in the NADH activity pathway, which was downregulated in IVM oocytes in our study (Supplementary Fig. 1). This suggests that IVM may compromise mitochondrial function, leading to impaired ATP production, increased oxidative stress, and ultimately impacting developmental competence (Van Blerkom 2011).

IVM also affected key epigenetic regulation, leading to distinct DNA methylation patterns in oocytes compared to IVO. Our results demonstrated that the global methylation level of IVM and IVO oocytes derived from antral follicles is approximately 40%. This finding is consistent with data reported previously, which showed a similar DNA methylation level in fully grown bovine oocytes (120 μm) (Barbosa Latorraca *et al.* 2023). We found significant hypomethylation in CpG islands and gene bodies in IVM oocytes compared to IVO. More specifically, hypermethylated tiles showed reduced methylation following IVM. This coincides with previous experiments using reduced representation bisulfite sequencing (RRBS), demonstrating that bovine IVM oocytes display global DNA hypomethylation compared to IVO oocytes (Jiang *et al.* 2018). However, in humans, no differences in DNA methylation of global DNA, promoters, gene bodies, and CpG islands are observed after IVM compared to IVO (Ye *et al.* 2020). One possible explanation for the differences between our findings and those of the human single-cell study is its limited sample size: only eight IVO oocytes from six patients and seven IVM oocytes from distinct patients. This small sample may constrain the generalizability of their results, given the variability in methylation patterns between individuals and oocytes.

In mammalian oocytes, DNA methylation at the promoter level has been negatively correlated with gene expression, whereas genes with high methylation levels at gene bodies are more likely to be expressed (Ivanova *et al.* 2020). We observed a trend of negative correlation between promoter methylation and gene expression, which was similar for IVM and IVO oocytes. In the comparison between IVM and IVO oocytes, pathway analysis for the hypermethylated (≥80%) DMRs at gene bodies revealed enrichment in DNA repair, macromolecule catabolic process, protein localization, enzyme inhibitor activity, and ATP binding. These pathways have been associated with key developmental events such as fertilization and zygote genome activation (Ruddock-D’Cruz *et al.* 2008, Zhao *et al.* 2023). As DNA methylation levels remain stable and similar to oocyte methylation levels across bovine cleavage stages (Ivanova *et al.* 2020), we hypothesize that the distinct hypermethylation patterns at the gene body between IVM and IVO oocytes may establish epigenetic marks influencing critical developmental pathways. As such, differences in IVM conditions could result in specific epigenetic modifications affecting gene regulation and cellular function during early embryonic development. However, further research is required to validate these findings.

Perhaps the most notable finding of this study is the imprinted gDMRs that were differentially methylated between IVO and IVM oocytes and include the maternally imprinted genes *NNAT*, *MEST* (also known as *PEG1*), and *SNRPN* (Fig. 3J). Loss of imprinting of these genes has been previously associated with the large offspring syndrome in cattle following ART, but also in the human analog, Beckwith–Wiedemann syndrome (Chen *et al.* 2015). Our results showed a tendency toward loss of methylation in genomic imprinting regions following IVM for *NNAT* and *MEST*. Interestingly, *MEST* has been found to exhibit higher methylation levels in IVO oocytes from nonstimulated compared to stimulated cows (Lopes *et al.* 2022), suggesting that the methylation status in our experiment is likely influenced by the maturation environment rather than the hormonal stimulation protocol. However, differences in superovulation protocols between studies may account for the discrepancies in findings. Therefore, the potential effect of superovulation cannot be entirely ruled out.

We also identified loss of methylation in *MIMT1*, which is a nonprotein-coding gene forming the imprinted paternally expressed gene 3 (*PEG3*) domain (Flisikowski *et al.* 2012). In bovine, alteration in *MIMT1* methylation has been associated with aberrant placental transcriptome and stillbirth (Flisikowski *et al.* 2012). Interestingly, the maternally imprinted insulin-like growth factor 2 (*IGF2*) and the paternally imprinted insulin-like growth factor 2 receptor (*IGFR2*) exhibited an unexpected methylation profile. The DNA methylation mark of *IGF2* and *IGF2R* was similar between IVM and IVO oocytes. However, *IGF2* displayed lower methylation levels than anticipated, and *IGF2R* exhibited higher methylation levels than usual for their respective imprinting statuses (Fig. 3J). A similar finding has been observed in another bovine study, where the *IGF2* gene exhibited higher levels of methylation in sperm compared to oocytes, while the *IGF2R* gene showed increased methylation in oocytes relative to sperm (Jiang *et al.* 2018). This differential methylation pattern suggests that imprinting regulation of these genes might involve other epigenetic mechanisms.

Interestingly, oocytes that underwent a combination of *in vivo* and IVM (VV group) exhibited altered gene expression profiles. The distinct transcriptomic profiles observed in VV, IVO, and IVM oocytes highlight the profound influence of maturation conditions on oocyte gene expression. Although we identified DEGs shared among the comparisons (13 DEGs between VV vs IVO and IVM vs IVO, 14 DEGs between VV vs IVM and IVO vs IVM, and 18 DEGs common to all comparisons), the expression profile did not conclusively indicate whether VV oocytes are more similar to IVO or IVM oocytes. We found common GO pathways enriched among VV vs IVM and VV vs IVO comparisons, including cellular response to stress, cellular catabolic processes, transcription corepressor activity, and ubiquitin protein ligase binding. Moreover, the DEGs between VV and IVO oocytes were enriched in stress response and metabolic process genes. These pathways were enriched in human MI oocytes rescued from stimulated cycles and matured *in vitro* compared to IVO oocytes (Jones *et al.* 2008, Hao *et al.* 2017). In human clinical settings, the rescue of immature oocytes in stimulated cycles is not a routine procedure since the developmental competence of these oocytes is significantly lower compared with IVO MII oocytes (Ferrer-Vaquer *et al.* 2019). These data suggest again that the suboptimal conditions *in vitro*, compared to *in vivo*, might have interfered with post-transcriptional modifications, transcript degradation, and selective translation that occur during maturation (Grondahl *et al.* 2013, Hu *et al.* 2022, An *et al.* 2024). Among the DEGs from IVM vs VV, we identified a cohort of maternal-effect genes, including *BMP15*, *PMS1*, and *UBE2A*, downregulated in VV oocytes, and *KPNA6* and *DMAP1*, upregulated in VV oocytes. These genes are involved in cumulus cell expansion, DNA repair, fertilization, and maintaining embryonic cell pluripotency (Galloway *et al.* 2000, Juengel & McNatty 2005, Fazzio *et al.* 2008, Rother *et al.* 2011), thus important for oocyte developmental competence. Among the DEGs from IVO vs VV, we identified two genes of the METTL family (*METTL4* and *METTL26*) downregulated in the VV group, which are involved in embryonic stem cell renewal (Wang *et al.* 2014, Zhang *et al.* 2020), while genes related to mitochondrial complex I (including *NDUFA13, NDUFB2,* and *NDUFB3*) involved in the oxidative phosphorylation electron transport chain (Padavannil *et al.* 2022), were downregulated in IVM oocytes.

The developmental competence of an oocyte hinges significantly upon the intricate functions of cumulus cells surrounding it. Cumulus cells are pivotal in nurturing and supporting the oocyte’s maturation process. To depict a complete overview of how the IVM protocol influences oocyte developmental competence, we performed transcriptome analysis on cumulus cells from IVM and IVO oocytes, resulting in 844 DEGs. The most abundant cluster of downregulated genes in IVM cumulus cells is related to the electron transport chain and mitochondrial respiratory chain complex I (Supplementary Fig. 4). This parallels findings in IVM oocytes, where pathways involved in NADH production were similarly affected. Thus, both cumulus cells and oocytes undergo transcriptional changes during IVM, emphasizing the interconnected effects of maturation conditions on cellular metabolism and function. It is important to highlight the significance of oxidative phosphorylation, the intracellular process responsible for generating the majority of the cell’s ATP through the electron transfer chain (Cannino *et al.* 2007). Cumulus cells can supply energy metabolites and ATP to the oocyte through gap junctions, influencing oocyte fertilization potential (Tamassia *et al.* 2004). Given the crucial functions of each mitochondrial electron transport chain complex, any alterations in one or more of these components are likely to disrupt the electron transport chain, leading to changes in ATP levels.

Taken together, our results demonstrate that IVM influences both the gene expression and DNA methylation profile of bovine oocytes, alongside changes in mitochondrial activity in oocytes and cumulus cells. These alterations in the transcriptome and epigenome indicate potential compromises in oocyte maturation and developmental potential. Future studies should focus on functional validation of the observed methylome modifications, particularly the hypomethylation of CpG islands and imprinted genes such as NNAT and MEST, to determine their direct impact on zygotic genome activation and embryo development. Moreover, targeted interventions to address mitochondrial dysfunction, such as optimizing culture conditions or restoring mitochondrial activity, could offer valuable insights for enhancing IVM protocols. This study lays the groundwork for future research to refine IVM practices and safeguard oocyte quality, ultimately advancing assisted reproductive technologies.

## Supplementary materials





## Declaration of interest

The authors declare that there is no conflict of interest that could be perceived as prejudicing the impartiality of the research reported.

## Funding

This research was supported by the European Union’s Horizon 2020 research and innovation program under the Marie Skłodowska-Curie grant agreement No 860960, and by Bijzonder Onderzoeksfonds GOA (Geconcerteerde Onderzoeksacties) 2018000504 (GOA030-18 BOF). KCP was supported by the Research Foundation Flandershttps://doi.org/10.13039/501100003130 (FWO) (grant numbers: 1228821N and 12A2H24N).

## Author contribution statement

CB, AVS, KS, and KP have designed the study. CB performed all experiments and wrote the original draft. NA, AFM, TDC, and OBP helped with the oocyte collection. CG and AG worked on the library construction. KS, AVS, KP, TF, EM, FVN, AG, GK, OBP, YG, and CG helped with the review and editing of the manuscript. SA, YG, and FC performed the formal analysis.

## Data availability

Raw data supporting the findings of this study were deposited in the National Center for Biotechnology Information (NCBI) Gene Expression Omnibus (GEO) database with accession number GSE291722.
