# Supplementary material for: Single-cell multiomic analysis reveals methylome and transcriptome deviations following oocyte maturation in vitro
Source: Reproduction. 2025 Jul 10;170(2):e250011. doi: 10.1530/REP-25-0011 (PMC12257493; doi:10.1530/REP-25-0011)
Supplement: Supplementary file 1 [file supplementary_materials.pdf]

A

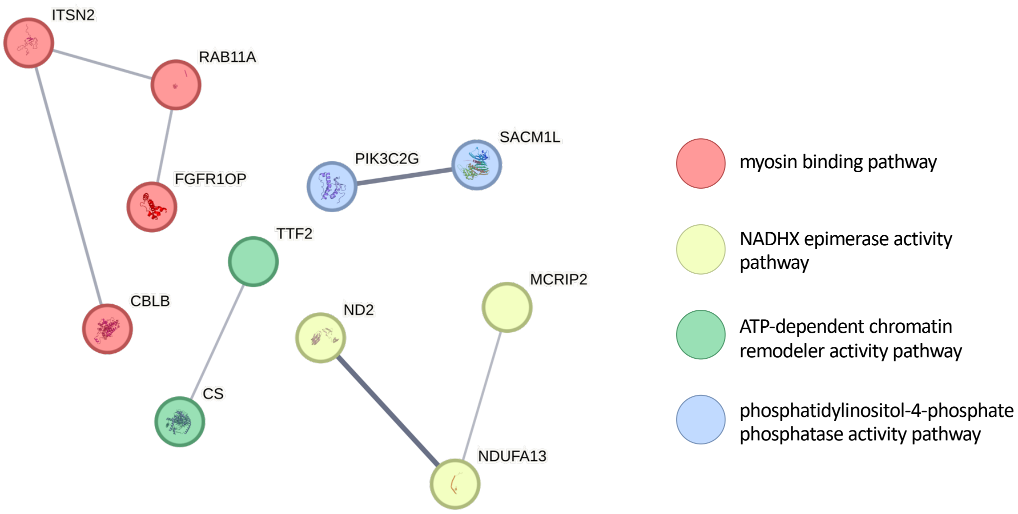

B

I

GO:MF		stats	
Term name	Term ID	p_{adj}	$-\log_{10}(p_{adj})$
myosin V binding	GO:0031489	2.448×10^{-2}	
microtubule binding	GO:0008017	7.537×10^{-2}	
GTP binding	GO:0005525	7.537×10^{-2}	
GTPase activity	GO:0003924	7.537×10^{-2}	

II

GO:MF		stats	
Term name	Term ID	p_{adj}	$-\log_{10}(p_{adj})$
NADHX epimerase activity	GO:0052856	2.030×10^{-3}	
NADH dehydrogenase activity	GO:0003954	3.680×10^{-3}	
electron transfer activity	GO:0009055	5.301×10^{-3}	

III

GO:MF		stats	
Term name	Term ID	p_{adj}	$-\log_{10}(p_{adj})$
citrate (Si)-synthase activity	GO:0004108	1.861×10^{-3}	
ATP-dependent chromatin remodeler activity	GO:0140658	3.254×10^{-2}	

IV

GO:MF		stats	
Term name	Term ID	p_{adj}	$-\log_{10}(p_{adj})$
phosphatidylinositol-4-phosphate phosphatase activity	GO:0043812	1.692×10^{-3}	
phosphatidylinositol phosphate 4-phosphatase activity	GO:0034596	1.974×10^{-3}	
phosphatase activity	GO:0016791	3.000×10^{-2}	

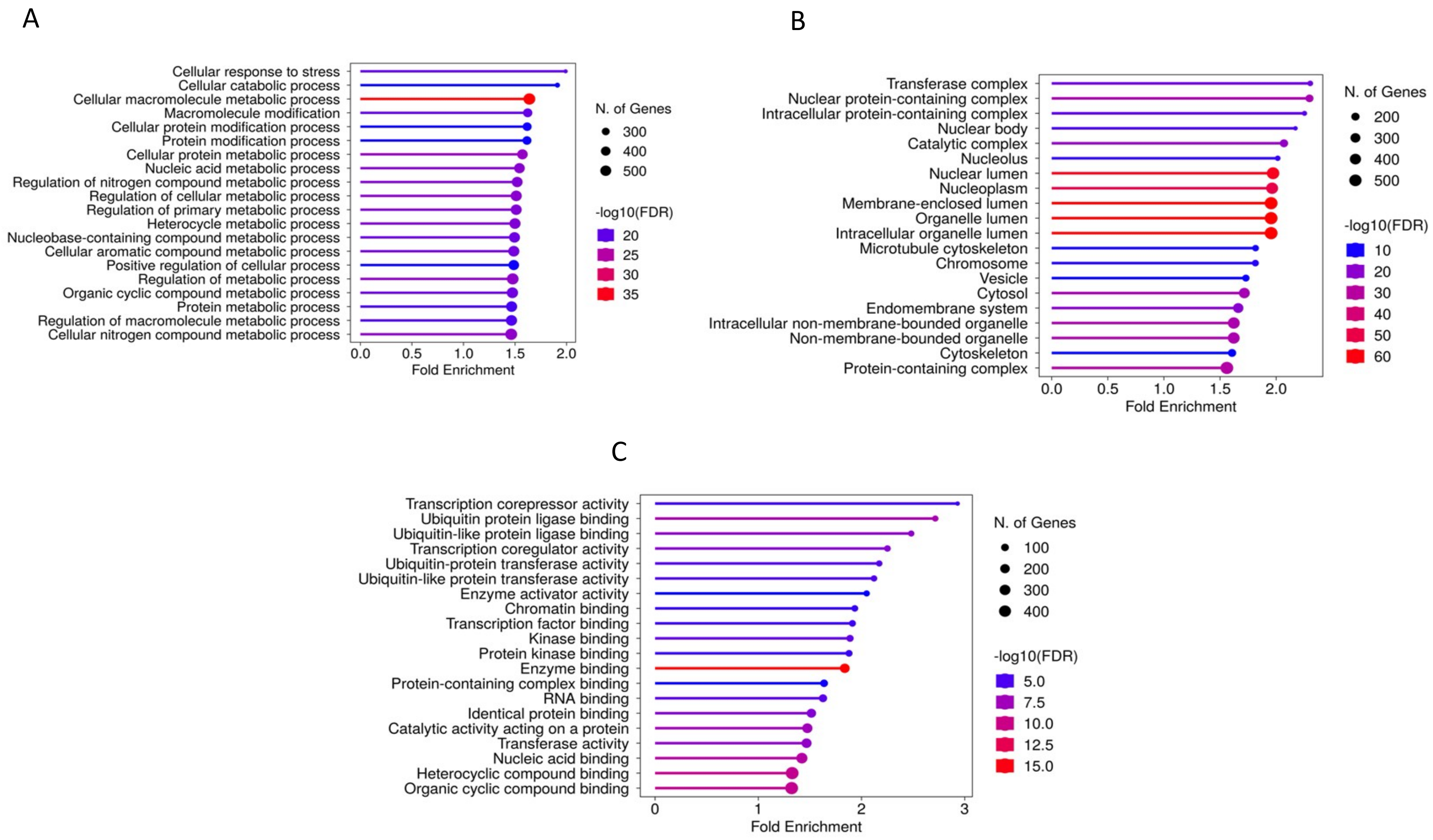
A
Cellular response to stress
Cellular catabolic process
Cellular macromolecule metabolic process
Macromolecule modification
Cellular protein modification process
Protein modification process
Cellular protein metabolic process
Nucleic acid metabolic process
Regulation of nitrogen compound metabolic process
Regulation of cellular metabolic process
Regulation of primary metabolic process
Heterocycle metabolic process
Nucleobase-containing compound metabolic process
Cellular aromatic compound metabolic process
Positive regulation of cellular process
Regulation of metabolic process
Organic cyclic compound metabolic process
Protein metabolic process
Regulation of macromolecule metabolic process
Cellular nitrogen compound metabolic process
0.0 0.5 1.0 1.5 2.0
Fold Enrichment
N. of Genes
300
400
500
-log10(FDR)
20
25
30
35
B
Transferase complex
Nuclear protein-containing complex
Intracellular protein-containing complex
Nuclear body
Catalytic complex
Nucleolus
Nuclear lumen
Nucleoplasm
Membrane-enclosed lumen
Organelle lumen
Intracellular organelle lumen
Microtubule cytoskeleton
Chromosome
Vesicle
Cytosol
Endomembrane system
Intracellular non-membrane-bounded organelle
Non-membrane-bounded organelle
Cytoskeleton
Protein-containing complex
0.0 0.5 1.0 1.5 2.0
Fold Enrichment
N. of Genes
200
300
400
500
-log10(FDR)
10
20
30
40
50
60
C
Transcription corepressor activity
Ubiquitin protein ligase binding
Ubiquitin-like protein ligase binding
Transcription coregulator activity
Ubiquitin-protein transferase activity
Ubiquitin-like protein transferase activity
Enzyme activator activity
Chromatin binding
Transcription factor binding
Kinase binding
Protein kinase binding
Enzyme binding
Protein-containing complex binding
RNA binding
Identical protein binding
Catalytic activity acting on a protein
Transferase activity
Nucleic acid binding
Heterocyclic compound binding
Organic cyclic compound binding
0 1 2 3
Fold Enrichment
N. of Genes
100
200
300
400
-log10(FDR)
5.0
7.5
10.0
12.5
15.0

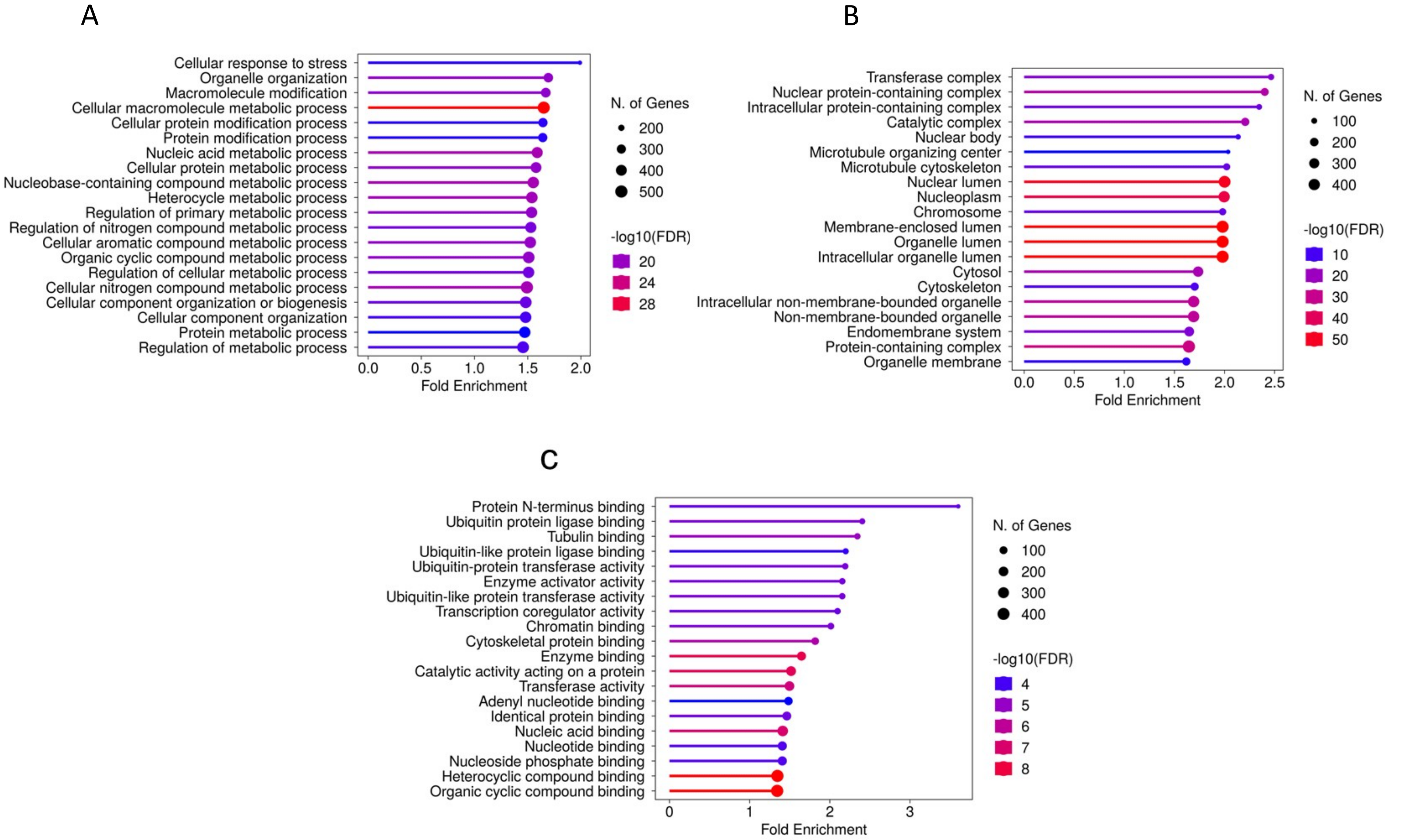
A
Cellular response to stress
Organelle organization
Macromolecule modification
Cellular macromolecule metabolic process
Cellular protein modification process
Protein modification process
Nucleic acid metabolic process
Cellular protein metabolic process
Nucleobase-containing compound metabolic process
Heterocycle metabolic process
Regulation of primary metabolic process
Regulation of nitrogen compound metabolic process
Cellular aromatic compound metabolic process
Organic cyclic compound metabolic process
Regulation of cellular metabolic process
Cellular nitrogen compound metabolic process
Cellular component organization or biogenesis
Cellular component organization
Protein metabolic process
Regulation of metabolic process
0.0 0.5 1.0 1.5 2.0
Fold Enrichment
N. of Genes
200
300
400
500
-log10(FDR)
20
24
28
B
Transferase complex
Nuclear protein-containing complex
Intracellular protein-containing complex
Catalytic complex
Nuclear body
Microtubule organizing center
Microtubule cytoskeleton
Nuclear lumen
Nucleoplasm
Chromosome
Membrane-enclosed lumen
Organelle lumen
Intracellular organelle lumen
Cytosol
Cytoskeleton
Intracellular non-membrane-bounded organelle
Non-membrane-bounded organelle
Endomembrane system
Protein-containing complex
Organelle membrane
0.0 0.5 1.0 1.5 2.0 2.5
Fold Enrichment
N. of Genes
100
200
300
400
-log10(FDR)
10
20
30
40
50
C
Protein N-terminus binding
Ubiquitin protein ligase binding
Tubulin binding
Ubiquitin-like protein ligase binding
Ubiquitin-protein transferase activity
Enzyme activator activity
Ubiquitin-like protein transferase activity
Transcription coregulator activity
Chromatin binding
Cytoskeletal protein binding
Enzyme binding
Catalytic activity acting on a protein
Transferase activity
Adenyl nucleotide binding
Identical protein binding
Nucleic acid binding
Nucleotide binding
Nucleoside phosphate binding
Heterocyclic compound binding
Organic cyclic compound binding
0 1 2 3
Fold Enrichment
N. of Genes
100
200
300
400
-log10(FDR)
4
5
6
7
8

A

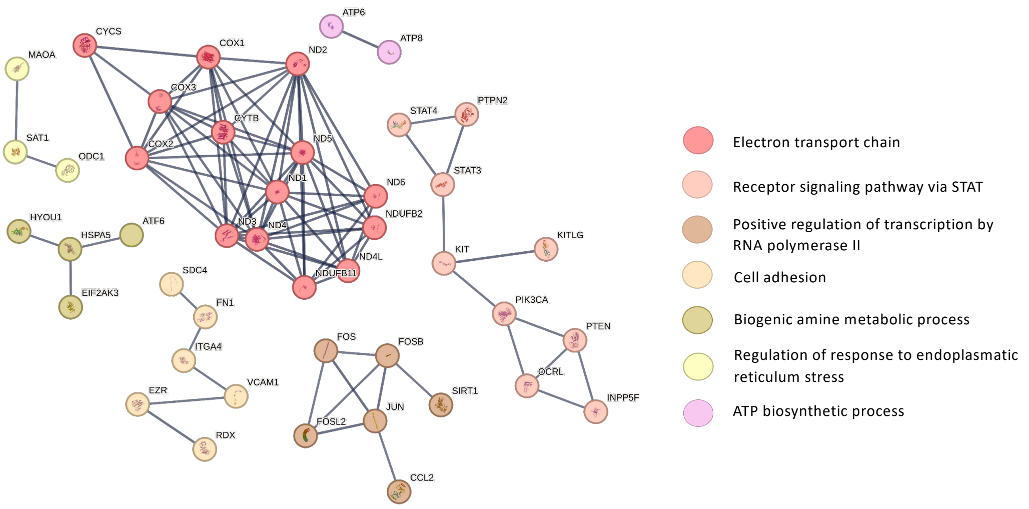

B

I

Term name	Term ID	p_{adj}
electron transport chain	GO:0022900	1.326×10^{-21}
mitochondrial respiratory chain complex I assembly	GO:0032981	1.587×10^{-14}
mitochondrial electron transport, NADH to ubiquinone	GO:0006120	3.430×10^{-14}

II

Term name	Term ID	p_{adj}
receptor signaling pathway via STAT	GO:0097696	9.862×10^{-7}
phosphate-containing compound metabolic process	GO:0006796	2.180×10^{-5}
reproduction	GO:0000003	5.749×10^{-3}
regulation of programmed cell death	GO:0043067	7.046×10^{-3}
negative regulation of primary miRNA processing	GO:2000635	8.260×10^{-3}

III

Term name	Term ID	p_{adj}
positive regulation of transcription by RNA polymerase II	GO:0045944	7.722×10^{-4}
response to growth factor	GO:0070848	7.722×10^{-4}
cellular response to oxidative stress	GO:0034599	9.317×10^{-4}
regulation of developmental process	GO:0050793	9.115×10^{-3}

IV

Term name	Term ID	p_{adj}
cell adhesion	GO:0007155	1.982×10^{-5}
integrin-mediated signaling pathway	GO:0007229	4.695×10^{-3}
cell junction organization	GO:0034330	5.982×10^{-3}

V

Term name	Term ID	p_{adj}
biogenic amine metabolic process	GO:0006576	1.948×10^{-6}

VI

Term name	Term ID	p_{adj}
regulation of response to endoplasmic reticulum stress	GO:1905897	2.391×10^{-8}
protein folding	GO:0006457	4.633×10^{-3}
post-translational protein targeting to membrane, transloca...	GO:0031204	6.078×10^{-3}

VII

Term name	Term ID	p_{adj}
ATP biosynthetic process	GO:0006754	1.742×10^{-4}
proton transmembrane transport	GO:1902600	4.134×10^{-4}
